# Tænia Solium

**Published:** 1870-01

**Authors:** D. C. Davies

**Affiliations:** Columbus, Wis.


ARTICLE V.—*Tænia Solium.* By D. C. DAVIES, M.D., Columbus, Wis.

IN the JOURNAL for December, 1869, we notice two cases of Tænia Solium, reported by Dr. Owen, of St. Luke's Hospital, in which that learned physician seems to manifest a decided preference for pumpkin seed, as an ejective agent in cases of tape worm. It is not our desire to differ with Dr. Owens, for the sake of being contrary, but from higher motives—that of elevating our profession in all its several departments, while in pursuit of truth, be that even in the simple treatment of tape worm. We have found the ætherial extract of male fern, in all cases where we have had occasion to use it, answering our highest expectations, as the following cases will illustrate:

CASE 1.—Mrs. R., æt. 35, a native of Wales, who, upon first appearance, would lead a physician, owing to her ænemic, cadaverous and nervous condition, to dream of some uterine affection. Few questions, however, soon dispelled such conclusion, as the following group of symptoms will show: Bowels irregular and sometimes accompanied with a sort of gnawing sensation—appetite capricious—sleep disturbed—unaccountably nervous and fretful—despondency, almost bordering on hypochondriasis—and occasionally voiding "pieces of some white stuff" with the fæces, which, after due examination, we found to be segments of tænia solium. Satisfied as to the correctness of our diagnosis we ordered the patient to abstain from all food until further orders; and prescribed the following draught, to be taken before retiring, and the same the following morning:

℞ Extracti filicis ætheris, - - - - ʒj ss;

S.—To be taken in a cupful of fresh milk.

At 10, A. M., the following day we ordered the annexed draught:

℞ Ol. ricini, - - - - - - - ℥j;
Ol. Terebinthinæ, - - - - - - ʒj;
M.

Calling again, some three hours later, we found she had free evacuations, containing several pieces of tape from four to six inches long, and a large number of segments. Not satisfied with the appearance of things, we gave the following powder:

℞ Pulv. Scammon.
Calomelanos, - - - - - aa gr. xij;
Pulv. Gambogæ, - - - - - gr. vj;
M.

In less than an hour from the taking of the powder the balance of the tape was expelled, measuring twenty-seven feet intact. This specimen we have preserved.

CASE 2.—Mr. O'R., æt. 42, a native of Ireland. Placed him under the same treatment, with this exception, that we gave him ʒij of the extract in each dose, and also double the powder, believing in the old axiom that purgatives would not kill an Irishman. The tape was ejected in half an hour after the powder was taken, but I failed to preserve this specimen intact, owing, I

suppose, as stated by the patient, to the "d — n crather coming through in sich a divil of a hurry." Neither of the pieces would measure over ten inches, but from the number we should think it would measure from thirty to forty feet.

It may be surmised that the castor oil and turpentine, or the powder alone, would have ejected the tape, but that could not have been so in the last case, as he had, previous to my seeing him, taken enormous doses of calomel, jalapa and gamboge, without any other effect than catharisis and the ejection of some segments of the tape.

In conclusion, we will venture one remark on Dr. Owens' treatment, to wit: That if he had prescribed the oil of the male fern in larger doses we think he would have met with better success. We believe in the most active treatment in the ejection of all such "varmints."

